# Advanced colorectal polyps with the molecular and morphological features of serrated polyps and adenomas: concept of a ‘fusion’ pathway to colorectal cancer

**DOI:** 10.1111/j.1365-2559.2006.02466.x

**Published:** 2006-03-01

**Authors:** J R Jass, K Baker, I Zlobec, T Higuchi, M Barker, D Buchanan, J Young

**Affiliations:** 1 Department of Pathology, McGill University Montreal, Canada; 2 Department of Surgical Oncology, Graduate School, Tokyo Medical and Dental University Tokyo, Japan; 3 Queensland Institute of Medical Research Brisbane, Australia

**Keywords:** adenoma, colon, hyperplastic, polyp, serrated

## Abstract

Jass J R, Baker K, Zlobec I, Higuchi T, Barker M, Buchanan D & Young J (2006) *Histopathology* **49**, 121–131

**Advanced colorectal polyps with the molecular and morphological features of serrated polyps and adenomas: concept of a ‘fusion’ pathway to colorectal cancer**

**Aim:**

To establish and explain the pattern of molecular signatures across colorectal polyps.

**Methods and results:**

Thirty-two sessile serrated adenomas (SSA), 10 mixed polyps (MP), 15 traditional serrated adenomas (SA), 49 hyperplastic polyps (HP) and 84 adenomas were assessed for mutation of *KRAS* and *BRAF* and aberrant expression of p53. The findings were correlated with loss of expression of O-6-methylguanine DNA methyltransferase (MGMT). *KRAS* mutation occurred more frequently (26.5%) than *BRAF* mutation (4.8%) in adenomas (*P* < 0.001) and particularly in adenomas with villous architecture (50%). Loss of expression of MGMT correlated with *KRAS* mutation in small tubular adenomas (*P* < 0.04). *BRAF* mutation was frequent in HPs (67%) and SSAs (81%), while *KRAS* mutation was infrequent (4% and 3%, respectively). Of MPs and SAs, 72% had either *BRAF* or *KRAS* mutation. Aberrant expression of p53 was uncommon overall, but occurred more frequently in MPs and SAs (12%) than adenomas (1%) (*P* < 0.04) and there was concordant loss of expression of MGMT.

**Conclusions:**

Molecular alterations that are characteristic of the serrated pathway and adenoma–carcinoma sequence can co-occur in a minority of advanced colorectal polyps that then show morphological features of both pathways. These lesions account for only 2% of colorectal polyps, but may be relatively aggressive.

## Introduction

The traditional view of epithelial polyps of the colorectum envisages a larger non-neoplastic category of hyperplastic polyps (HPs) that can be safely ignored and a smaller category of neoplastic polyps or adenomas that are precancerous.^1^ With respect to adenomas, it is clear that the majority will not progress to colorectal cancer (CRC).^2^ The risk of cancer developing within an adenoma increases with size, grade of dysplasia (synonymous with intraepithelial neoplasia) and villosity.^3^ These features, together with polyp numbers, are also predictive of metachronous neoplasia and may therefore influence the decision to offer follow-up endoscopic surveillance.^4^ However, the grading of dysplasia and estimation of the extent of villous change may not be fully reproducible and it may be difficult to distinguish new from recurrent adenomas. Establishing a risk profile on the basis of the traditional features of adenoma is therefore not always straightforward.

The addition of molecular profiling to polyp description offers hope of a more objective and therefore reproducible approach to the classification of colorectal adenomas. The genetic evolutionary paradigm envisages a linear sequence of changes beginning with bi-allelic inactivation of *APC*, followed by oncogenic *KRAS* mutation and culminating in inactivation of *TP53* at the transition from adenoma to carcinoma.^5^ While there is good evidence that *KRAS* mutation is associated with advanced adenoma features^6^ and could therefore be used as objective evidence of aggression, this approach has a number of limitations. First, *KRAS* mutation also occurs frequently in dysplastic aberrant crypt foci (microadenomas) and in some small tubular adenomas, suggesting that *KRAS* mutation may initiate a subset of small adenomas with limited potential for progression.^6^,^7^ Second, around 70% of CRCs lack mutation of *KRAS*.^8^ From this it follows that most of the precancerous lesions that eventually become cancers do not in fact have *KRAS* mutation. There is increasing evidence that CRC evolves through a number of pathways and the traditional adenoma–carcinoma sequence, with its accompanying genetic steps, provides a surprisingly narrow window of understanding of this multipathway reality.^9^

In recent years, the fundamental division of colorectal polyps into precancerous adenomas and innocent HPs has begun to erode and the concept of an alternative serrated pathway has gained support. This revision began with the description of an intermediate lesion described as serrated adenoma (SA).^10^ Initially, however, SA was not conceived as an intermediate category of polyp but essentially as an adenoma with a superimposed serrated architecture that conferred only a superficial likeness to a HP.^10^ Additionally, the mixed polyp (MP) was perceived as a chance collision between a HP and an adenoma, giving a combined polyp. These preliminary interpretations did not represent a major departure from the traditional classification of colorectal polyps but preserved the fundamental distinction of neoplastic adenomas versus non-neoplastic HPs.

Subsequently, it was argued that HP and SA are related lesions.^11^ The latter could arise within the former (giving a MP) or could develop as *de novo* SA but through mechanisms shared with HPs. This proposal subsequently received strong support through the demonstration of molecular alterations common to both types of serrated polyp, notably mutation of *BRAF* and extensive DNA methylation.^12^ This viewpoint was consolidated through the formal recognition of two largely independent pathways of colorectal tumorigenesis: (i) the traditional adenoma–carcinoma sequence associated with chomosomally unstable CRCs,^13^ and (ii) the ‘serrated pathway’ culminating in CRCs with DNA microsatellite instability (MSI), mutation of *BRAF* and extensive DNA methylation.^12^,^14^–^20^

This paper explores the possibility that the early evolution of colorectal cancer is not limited to two essentially independent pathways, but often combines components of these pathways. Indeed, the successful ‘fusion’ of the hyperproliferation and crypt fission that characterize adenomas^21^ with the inhibition of apoptosis that has been linked with serrated polyps^22^,^23^ may generate lesions with enhanced aggressiveness. Specifically, it is suggested that methylation of the DNA repair gene *O-6-methylguanine DNA methyltransferase* (*MGMT*), mutation of *KRAS* and inactivation of *TP53* provide critical combinations of molecular ‘cross-over’ between the two pathways that occur at the stage of precancerous polyps.

## Materials and methods

### Tissues

The colorectal polyps used in this study were from a previously characterized subset of a consecutive series of 1250 colonoscopically derived lesions.^24^ The study group included all SAs (sessile and traditional) and MPs and a subset of conventional adenomas and HPs. The previous study analysed proliferative indices and there was therefore selection of HPs that were large and well-oriented and likely to provide sufficient numbers of longitudinally sectioned crypts. Polyps were eliminated from the present study if there was insufficient residual tissue, blocks contained more than one polyp, polyps contained foci of invasive cancer, or there was failure of DNA amplification for both the *BRAF* and *KRAS* mutation assays. Fifteen additional polyps from the parent series generated a total study group of 190 polyps. These were reviewed by the first author and classified as: HPs (*n* = 49), tubular adenomas (TAs) (*n* = 62), tubulovillous and villous adenomas (TVAs/VAs) (*n* = 22), sessile SAs (SSAs) (*n* = 32), MPs (*n* = 10) and traditional SAs (*n* = 15). SSA has also been termed sessile serrated polyp and serrated polyp with atypical proliferation.^17^,^25^–^27^ SSA is distinguished from HP on the basis of greater size, aberrant architecture, atypical proliferation, hypermucinous epithelium, predilection for proximal colon^25^ and molecular features including a higher frequency of *BRAF* mutation and more extensive DNA methylation.^14^,^16^,^17^ However, the combination of architectural and cytological changes is insufficient for a diagnosis of dysplasia. Serrated polyps with dysplasia include MPs and SAs. MPs may be conceived as a combined lesion that includes: (i) a serrated component that is non-dysplastic and resembles HP or SSA, and (ii) a dysplastic component that may resemble either SA or conventional adenoma.^28^ For the purposes of this study, lesions were also included as MPs if they comprised separate serrated and non-serrated components that were both dysplastic. SAs are homogeneous lesions in which there is epithelial serration reminiscent of a HP or SSA together with architectural and cytological changes warranting a diagnosis of dysplasia. The cytological atypia of SA may appear similar to that of conventional adenoma in which nuclei are elongated, hyperchromatic and pseudostratified. However, some SAs may be characterized by non-adenomatous forms of dysplasia in which the nuclei are enlarged, ovoid, vesicular and contain a prominent nucleolus, while the cytoplasm is relatively abundant and eosinophilic. The anatomical site of polyps was grouped as proximal colon (up to splenic flexure) versus distal colon and rectum combined. The study was approved by the Institutional Review Board of the Faculty of Medicine, McGill University.

### Dna extraction

Genomic DNA was extracted from archival paraffin-embedded tissue sections using DNeasy Tissue Kit (Qiagen, Hilden, Germany) according to the manufacturer's instructions except for the omission of xylene treatment. Briefly, five 4-µm non-microdissected sections (polyps were generally accompanied by little normal mucosa) and 180 µl of ATL buffer were dissolved at 80°C and then digested overnight with 20 µl of proteinase K at 55°C. Following centrifugation at 2000 r.p.m. (300 ***g***) for 5 min, the lower phase was applied to a DNeasy tissue column. Bound DNA was washed by centrifugation through ethanol and then eluted from the column using AE buffer.

### *KRAS* and *BRAF* mutations

*KRAS* mutation analysis at codons 12 and 13 was performed using direct automated sequencing of a fragment containing codon 12 and 13 in exon 1 of the *KRAS* gene, amplified using a touchdown polymerase chain reaction (PCR) cycle and hotstart protocol. PCR products were initially purified and then directly sequenced using BigDye version 3.1 dye terminators and an ABI 3100 DNA fragment analyser. The sequence at codon 12 and 13 was determined using Mutation Surveyor (SoftGenetics, State College, PA, USA) software.

*BRAF* mutation analysis at codon 600 (V600E; formerly V599E) was performed by a real-time PCR-based allelic discrimination method as previously described.^29^ Briefly, real-time PCR was performed using allele-specific primers designed to amplify selectively the wild-type (T1796) and mutant (A1796) *BRAF* alleles. The primer sequences were as follows: V, 5′-GTGATTTTGGTCTAGCTACtG*T*; E, 5′-CGCGGCCGGCCGCGGCGGTGATTTTGGTCTAGCTACcG*A*; AS, 5′-TAGCCTCAATTCTTACCATCCAC. PCR amplification and melting curve analysis were performed on a Rotor-gene 3000 (Corbett Research, NSW, Australia). Genomic DNA was amplified in a 15-µl volume containing 1 × Platinum SYBR Green qPCR SuperMix-UDG (Invitrogen, Carlsbad, CA, USA), forward primer V (300 nm), forward primer E (900 nm) and reverse primer AS (300 nm). The cycling conditions were as follows: 50°C for 2 min, 95°C for 2 min, 40 cycles of 95°C for 15 s and 60°C for 60 s. After amplification, samples were subjected to a temperature ramp from 60°C to 99°C, rising 1°C each step. For wild-type samples, single peaks were observed at 80°C while samples containing mutant alleles produced single peaks at 85°C.

### p53 immunohistochemistry and scoring

This was undertaken on all adenomas and all serrated polyps with dysplasia (traditional SA and MP). Most of these polyps had been immunostained previously for MGMT.^24^ Following deparaffinization and rehydration of 4-µm sections and antigen retrieval using ethylene diamine tetra-acetic acid and microwaving, the sections were subjected to peroxidase blockade (Dako EnVision bottle 1; Mississauga, Canada) and then incubated in 10% goat serum to minimize non-specific staining. They were subsequently incubated with the primary anti-p53 antibody **(**DO-7, from DakoCytomation, Mississauga, Canada) at a dilution of 1 : 100 for 60 min at 37°C. After washing, the sections were incubated with secondary antibody (EnVision bottle 2) for 30 min, washed again and then developed with the chromogen AEC for 30 min. Finally, the sections were counterstained with Gill's haematoxylin. Polyps were scored as showing loss of expression of MGMT if there was complete absence of nuclear expression throughout one or more crypts. Polyps were scored as positive for aberrant p53 expression if there were distinct subclones characterized by strong nuclear expression that implicated at least 50% of nuclei.

### Statistics

Data were analysed by χ^2^ test, Fisher's exact test or *T*-test, as appropriate, using SAS software, version 8.2 (Cary, NC, USA). *P*-values < 0.05 were interpreted as significant.

## Results

### *KRAS* and *BRAF* mutation

DNA failed to amplify in the *KRAS* assay for one SSA and one TA < 10 mm. Overall, 34 of 188 polyps (18%) had mutation of *KRAS*. Twenty-eight mutations were in codon 12 (20 G→A, seven G→T and one G→C) and six mutations were in codon 13 (all G→A). One serrated adenoma had two *KRAS* mutations in codon 12 (G→T at position 35 and T→G at position 36). *BRAF* mutation at V600E could be assessed in all polyps except for a single TA < 10 mm. *BRAF* mutation was found in 82 of 189 polyps (43%). *BRAF* and *KRAS* mutations were negatively correlated, with only four polyps having both mutations (two TAs, one TVA and one SSA). The three conventional adenomas with mutations of both *BRAF* and *KRAS* were among only four adenomas that had any *BRAF* mutations at all. Mutation frequencies for both *KRAS* and *BRAF* were distributed differently across the seven polyp groups (Table 1).

**Table 1:** Frequency of *KRAS* and *BRAF* mutation and loss of expression of O-6-methylguanine DNA methyltransferase (MGMT) by polyp type

Type of polyp	*KRAS* mutation	*BRAF* mutation	MGMT loss
Hyperplastic polyp	2/49 (4%)	33/49 (67%)	6/42 (14%)
Sessile serrated adenoma	1/31 (3%)	26/32 (81%)	7/31 (23%)
Serrated adenoma	4/15 (27%)	5/15 (33%)	2/15 (13%)
Mixed polyp	5/10 (50%)	4/10 (40%)	7/9 (78%)
Tubular adenoma < 10 mm	7/38 (18%)	2/38 (5%)	8/36 (22%)
Tubular adenoma > 10 mm	4/23 (17%)	0/23 (0%)	11/20 (55%)
Adenoma with villosity	11/22 (50%)	2/22 (9%)	7/22 (32%)

Mutation frequencies for both KRAS (*P* < 0.0001) and BRAF (*P* < 0.0001) are distributed differently across the seven classes of polyp (see Results for individual comparisons). Distribution of MGMT loss differs across the seven classes of polyp (*P* < 0.001).

Note: no result for KRAS in one sessile serrated adenoma (SSA) and one tubular adenoma (TA) or for BRAF in one TA. MGMT immunstaining not performed in 15 polyps (seven HPs, one SSA, one MP and six TAs).

*KRAS* mutation occurred in 26.5% and *BRAF* mutation in 4.8% of adenomas (all types) (Table 1) (*P* < 0.0001). TVAs/VAs were more likely to have *KRAS* mutation (50%) than TAs < 10 mm (18%) (*P* < 0.004) or TAS > 10 mm in diameter (17%) (*P* < 0.02). In the case of TAs there was a trend for *KRAS* mutation to occur more frequently in polyps from the proximal colon (*P* = 0.08) and in females (*P* = 0.07).

SSAs were more likely to have *BRAF* mutation (81%) than either SAs (33%) (*P* < 0.001) or MPs (40%) (*P* < 0.02). *KRAS* mutation was infrequent among both SSAs (3%) and HPs (4%) (Table 1). Patient age, gender and anatomical location were not predictors of *BRAF* mutation in either SSAs or HPs. The mean age of subjects with SSAs (64 years) differed from that of subjects with HPs (55 years) (*P* < 0.001).

**Figure 1:**
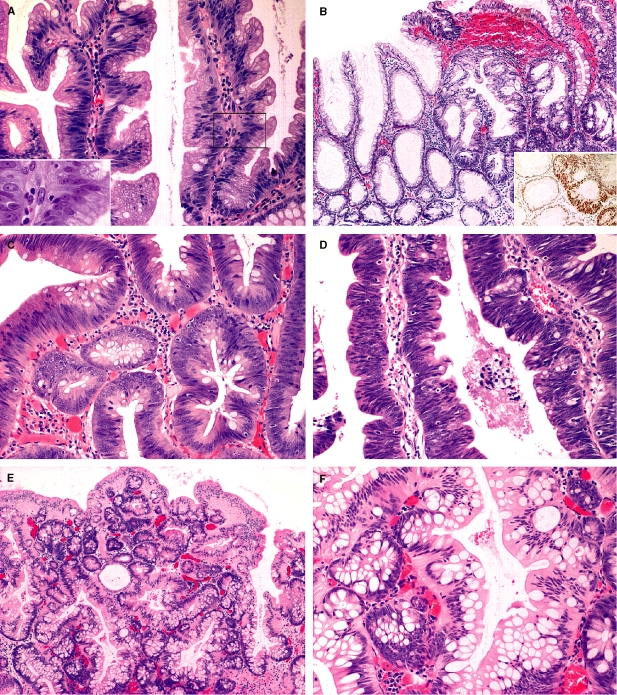
**A**, Serrated adenoma (SA) (*BRAF* mutation) with a ‘hyperplastic’ appearance but with architectural and cytological features of a non-adenomatous form of dysplasia. The latter include marked epithelial serration and surface papillarity and nuclei that are ovoid, vesicular and contain a prominent nucleolus (inset). The columnar cells (inset) contain apical mucin droplets, similar to sessile SA (SSA). **B**, Mixed polyp (*BRAF* mutation) comprising SSA (left) and SA with high-grade dyplasia showing back-to-back glands (right) and aberrant expression of p53 (inset). **C,D**, Two mixed polyps (MPs) (both SA/tubulo-villous adenoma and with *KRAS* mutation) in which the serrated epithelium has an adenomatous appearance as evidenced by elongated hyperchomatic nuclei with marked stratification and a dark amphophilic cytoplasm. The pure adenomatous component is not shown. **E,F**, Low- and medium-power images of a SA (*KRAS* mutation) in which complex microacini have resulted in markedly serrated epithelial contours. The epithelium comprises numerous goblet cells and absorptive-type columnar cells with eosinophilic cytoplasm and is reminiscent of the goblet cell variant of hyperplastic polyp. These examples illustrate the range of appearances and genetic changes that are encompassed by ‘traditional’ SA.

*BRAF* and *KRAS* mutations were distributed in roughly similar proportions across MPs (90% had either *BRAF* or *KRAS* mutation) and SAs (60% had either *BRAF* or *KRAS* mutation) (Table 1). The frequent finding of either *BRAF* or *KRAS* mutations in both types of serrated polyp indicated that MPs and SAs might be heterogeneous lesions. These 25 serrated polyps with dysplasia were therefore grouped differently. Group A polyps (*n* = 16) included a non-dysplastic serrated component and/or dysplastic epithelium in which the architectural and cytological changes were more reminiscent of HP than adenoma (Figure 1A,B). Group B polyps (*n* = 9) comprised serrated polyps in which the epithelial dysplasia appeared adenomatous (Figure 1C,D). *BRAF* mutation occurred in 10/16 Group A polyps but only 1/9 Group B polyps (*P* < 0.03). *KRAS* mutation occurred in only 3/16 Group A polyps but in 5/9 Group B polyps (*P* = 0.06). In each of the five Group B polyps with *KRAS* mutation, the adenomatous component showed both villous change and serration.

With respect to the 25 serrated polyps with dysplasia, only five occurred in the proximal colon (up to the splenic flexure). Two of these had *BRAF* mutation (both Group A) and two had *KRAS* mutation (both Group B). Seven of the 11 *BRAF* mutations occurred in polyps derived from the left colon or rectum (remaining two polyps with *BRAF* mutation from site unknown). The three Group A SAs with *KRAS* mutation (all from the left colon or rectum) comprised numerous goblet cells and adjacent cells with eosinophilic cytoplasm and no mucin production or microvesicular appearance (Figure 1E,F). These SAs therefore resembled the goblet cell variant of HP.

### Loss of expression of mgmt

Loss of expression of MGMT was distributed differently (*P* < 0.001) across the different types of polyps, being observed most frequently among MPs, TA > 10 mm and TVAs/VAs (Table 1). There was no correlation between loss of expression of MGMT and either *KRAS* or *BRAF* mutation across the full range of polyp types. However, among TAs < 10 mm, *KRAS* mutation occurred in 3/25 (12%) adenomas with no MGMT loss but in 4/8 (50%) adenomas with MGMT loss (*P* < 0.04). Since there were few *KRAS* mutations in this subset and most *KRAS* mutations (5/7) were G→A, it was not possible to demonstrate an association between MGMT loss and G→A mutation in *KRAS*.

### Aberrant expression of p53 and correlation with MGMT loss

**Figure 2:**
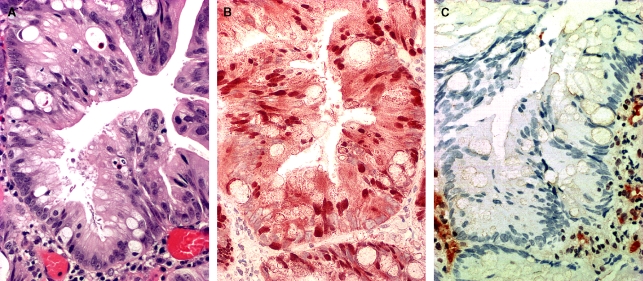
High-power field of a serrated adenoma with high-grade dysplasia (**A**) in which there is aberrant nuclear expression of p53 (**B**) and loss of nuclear expression of O-6-methylguanine DNA methyltransferase (C).

Sixty-two TAs, 22 TVAs/VAs, 15 SAs and 10 MPs were immunostained for p53. Weak expression of nuclear p53 occurred frequently within the proliferative compartment in all types of polyps and was ignored. Aberrant p53 expression was observed in only four polyps: one TA, one MP (Figure 1B) and two SAs (Figure 2). Overall, only 1/84 (1%) conventional adenomas showed aberrant expression of p53 compared with 3/25 (12%) serrated polyps with dysplasia (*P* < 0.04). In the latter, aberrant p53 expression coincided with high-grade dysplasia and reduced or complete loss of expression of MGMT (Figures 1B and 2). The TA with aberrant p53 expression had normal expression of MGMT.

## Discussion

The scientific basis for the prevention of cancer depends upon an understanding of early events in pathogenesis, including the mechanisms underlying initiation and growth of precancerous polyps. Recommendations for the management of patients with colorectal polyps currently recognize only a single principal type of clinically important polyp: the adenoma. The current study includes additional types of polyp that are likely to have malignant potential, namely serrated polyps with (MP and SA) and without (SSA) dysplasia. This discussion will focus first on adenomas, second on non-dysplastic serrated polyps and will then conclude with the concept that features of these two types of polyp can co-occur or become ‘fused’ in subtypes of advanced polyps with mixed cytomorphology (hyperplastic and dysplastic) and a serrated and/or villous architecture.

### Adenomas

The adenomas in this study were grouped as TAs < 10 mm, TAs > 10 mm and TVAs/VAs. Overall, *KRAS* mutation occurred in 26.5% of adenomas while *BRAF* mutation was detected in only 4.8%. Three of the four *BRAF* mutations occurred in adenomas that also had *KRAS* mutation. It is well established that *BRAF* and *KRAS* mutation rarely occur in the same colorectal neoplasm.^12^,^16^,^30^ Furthermore, *BRAF* mutations are much more typical of serrated polyps than adenomas.^14^,^31^ The assay for *BRAF* mutation used in this study was highly sensitive and it is possible that the mutation was being identified in normal mucosa included with the polyp. *KRAS* mutation may occur within apparently normal colorectal mucosa and the same could apply to *BRAF*.^32^ Therefore, the finding of *BRAF* mutation in a small subset of adenomas could be spurious and the true incidence of *BRAF* mutation in colorectal adenomas could be lower than 4.8%. *BRAF* mutation frequencies of 0%^14^ and 3%^31^ have been reported in other series of colorectal adenomas.

In previous studies of *KRAS* in colorectal adenomas, mutation has been associated negatively with flat and depressed TAs and positively with polypoid appearance, increasing size, dysplasia, villous change and synchronous colorectal cancer.^6^,^33^–^35^ In by far the largest study, which included 738 adenomas obtained from 639 participants in a dietary intervention trial, multivariate analysis showed that the independent predictors of *KRAS* mutation were age of subject, presence of villous architecture and high-grade dysplasia, but not size of adenoma.^6^ It is well known that adenoma size, dysplasia and villous architecture are interrelated and account has to be taken of this in assessing results. In this study *KRAS* mutation occurred with the same frequency in small (18%) and large (17%) adenomas but was significantly more frequent in adenomas that included a villous architecture (50%). A previous study showed a very high frequency of *KRAS* mutation (93%) in flat adenomas with a tubulovillous architecture.^34^ We would agree with the suggestion that *KRAS* mutation is linked with the development of villous change and does not influence adenoma growth in an independent manner.^6^ In this study, high-grade dysplasia was diagnosed only when it amounted to carcinoma *in situ*. This was observed in none of the 84 adenomas but in four serrated polyps, of which three showed aberrant expression of p53 and the fourth had *KRAS* mutation. Age was not a predictor of *KRAS* mutation in this study. However, many of the patients were undergoing continuing colonoscopic surveillance for colorectal polyps and it is known that after clearance of large polyps, time is required for the growth of newly initiated polyps.^36^ This could explain why adenomas were smaller in subjects aged > 60 years than in subjects aged < 60 years (data not shown).

### Non-dysplastic serrated polyps: HP and SSA

Non-dysplastic serrated polyps comprise conventional HPs and the variant lesion which has been described as SSA. In this study, SSAs accounted for approximately 3% of the overall series of polyps and were more age-related than HPs. Importantly, SSAs have been linked with the subset of colorectal cancer with *BRAF* mutation, DNA methylation, MSI and serrated architecture.^14^,^18^,^37^ In this study there was a high frequency of *BRAF* mutation in conventional HPs (67%) as well as in SSAs (81%). Previous reports have shown very similar results for *BRAF* mutation in SSA,^16^ but higher frequencies of *KRAS* mutation and lower frequencies of *BRAF* mutation in HPs.^12^,^16^,^38^ As mentioned in Materials and methods, there had been selection of larger HPs in an earlier cell kinetic study involving the same material. Large HPs are more likely to include the subset described as ‘microvesicular’, in which the columnar cells contain apical mucin droplets within small vesicles while goblet cells are rendered inconspicuous.^25^*BRAF* mutation occurs more frequently in the microvesicular variant of HP.^16^ By contrast, *KRAS* mutation occurs much more commonly in the goblet cell variant of HP, which is usually small, located in the left colon or rectum and deviates minimally from normal colorectal mucosa in terms of differentiation and architecture.^16^,^25^ The latter were under-represented in this series (details not shown).

### dysplastic serrated polyps: MP and SA

Serrated polyps with dysplasia, i.e. MPs and SAs, together comprised only 2% of the overall consecutive series of 1250 polyps. While mutation of *KRAS* and *BRAF* was associated with conventional adenoma and SSA, respectively (see above), *BRAF* and *KRAS* mutation occurred with similar frequency in both MPs (40% and 50%, respectively) and SAs (33% and 27%, respectively). In the literature, the frequency of *BRAF* and *KRAS* mutation in MP or SA has ranged from 36 to 100% and from 0% to 60%, respectively.^12^,^16^,^39^–^42^ These findings indicate that this subset of colorectal polyps is likely to be heterogeneous in terms of its molecular origins. These polyps were therefore reclassified according to their resemblance to HP or SSA (Group A) (Figure 1A,B,E,F) or to conventional adenoma (Group B) (Figure 1C,D). Particular histological features among the Group A polyps were: marked serration, a papillary or villous architecture, a relatively abundant eosinophilic cytoplasm, columnar cells with apical mucin droplets, nuclei that were enlarged, ovoid, vesicular and contained a prominent nucleolus, and adjacent non-dysplastic serrated polyp. Particular histological features among the Group B polyps were: some glandular serration, frequent villous change and epithelial dysplasia that appeared adenomatous (cytoplasmic basophilia and nuclei that were elongated, pseudostratified and hyperchromatic without a prominent nucleolus). Importantly, *BRAF* mutation occurred more frequently among Group A polyps (*P* < 0.03), whereas there was a trend for *KRAS* to be more frequent among Group B polyps (*P* = 0.06).

The preceding findings indicate that serrated polyps with dysplasia evolve through at least two independent histogenetic pathways. Group A polyps are likely to be initiated by *BRAF* mutation and are serrated lesions at the outset. Group B polyps may begin as conventional adenomas and then become serrated and villous following mutation of *KRAS*. There may be instances in which *KRAS* mutation can initiate lesions which are serrated at the outset and become dysplastic. While this suggestion remains speculative, it may apply to the Group A serrated polyps that show a distinct likeness to the goblet cell variant of HP in which columnar cells are eosinophilic and lack mucin-filled microvesicles and *KRAS* mutation is frequent (Figure 1E,F).^16^,^25^ The literature refers to the concept of ‘traditional’ SA.^43^ Based on the current findings, it is likely that several mechanisms can account for adenomatous lesions with glandular serration and that ‘traditional’ SA is not a single entity.

### Concept of ‘fusion’ pathways to crc

Colorectal polyps have traditionally been classified into distinct histogenetic types that may progress to CRC through independent pathways of colorectal tumorigenesis (Table 2). However, in addition to the two ‘classical’ pathways to CRC shown in Table 2, there may be ‘fusion’ pathways that combine mechanisms associated with both adenomas and serrated polyps. This would explain why many CRCs display phenotypes associated with serrated polyps as well as adenomas.^44^ Three possible examples of such fusion pathways are shown in Table 3. It is difficult to observe directly the actual point of transition from benign to malignant colorectal lesions. Once the key rate-limiting step is achieved it is likely that the transition to cancer occurs rapidly and the precursor lesion is then overtaken by the malignancy. Changes leading to inactivation of either *TP53*^5^ or the DNA mismatch repair gene *MLH1*^13^ are likely to be two such rate-limiting mechanisms. Only a single instance of loss of expression of MLH1 was observed in the present series of polyps and the adenoma in question was inferred to be from a patient with Lynch syndrome.^24^

**Table 2:** Concept of discrete colorectal lesions and progression to colorectal cancer via independent pathways

Initiation	Lesions	Methylation	Progression	Instability/ploidy
*KRAS*	ACF and HP	+	Rare	Not applicable
*BRAF*	SSA and SA	+++	MLH1*	Microsatellite/diploid
*APC*	TA	+/–	TP53*	Chromosomal/aneuploid

ACF, Aberrant crypt foci (hyperplastic or dysplastic); HP, hyperplastic polyp; SSA, sessile serrated adenoma; SA, serrated adenoma; TA, tubular adenoma.

* Inactivation of MLH1 and TP53 is associated with malignant progression.

**Table 3:** ‘Fusion’ pathways brought about by the sequential alteration of genes (linked to separate lesions and pathways in Table 2) and with the second alteration associated with a superimposed morphology

Fusion	Mechanism	Superimposed morphology	Instability
*BRAF-TP53*	Methylation *MGMT*	Dysplasia on serration	Various/subtle*
*APC-KRAS*	Methylation *MGMT*	Villosity on adenoma	Various/subtle*
*KRAS-APC*	Methylation *APC*	Dysplasia on serration	Various/subtle*

* MGMT inactivation predisposes to G:T mismatches and chromosomal instability through futile cycles of excision and repair as well as to mutation of KRAS and TP53.^9^ Partial methylation of MLH1 may also lead to low-level microsatellite instability.^37^

The interpretation of immunostaining for p53 is problematic insofar as increased expression of the wild-type protein occurs in areas of increased proliferation and must be distinguished from the diffuse and strong nuclear staining associated with retained mutant protein. However, several studies have described low frequencies of p53 expression in TAs with low-grade dysplasia^45^,^46^ and even in VAs.^47^ Conversely, there is general agreement that aberrant p53 expression is closely associated with the presence of high-grade dysplasia amounting to carcinoma *in situ*.^34^,^46^,^48^ Aberrant retention of presumed mutant nuclear p53 was rarely observed in the present series, although it occurred more frequently in serrated polyps with dysplasia (12%) than in adenomas (1%). One of the polyps with aberrant expression of p53 was a mixed polyp with *BRAF* mutation (Figure 1B). Had it not been removed, this polyp may have progressed within a short time frame to the subset of CRC with *BRAF* mutation, DNA methylation, *TP53* mutation and DNA microsatellite stable status (a ‘fusion’ pathway shown in Table 3).^49^,^50^

Loss of expression of the DNA repair gene *MGMT* is associated with methylation of the promoter region^45^,^51^,^52^ and the latter change has been linked causatively with G:C to A:T transition mutations in *TP53*.^53^ In the present study, complete or partial loss of expression of MGMT coincided with aberrant nuclear expression of p53 in three serrated polyps with dysplasia (Figure 2), but not in the single tubular adenoma with aberrant p53 expression. Only one previous study has attempted to correlate MGMT and p53 expression in colorectal polyps.^45^ In that study, 4.3% of adenomas showed aberrant p53 expression but none had loss of MGMT. It is possible that the link between *MGMT* silencing and *TP53* mutation is more evident in the serrated pathway than in the adenoma–carcinoma sequence. The frequency of *TP53* mutation in SAs has ranged from 5 to 50% in the literature.^39^,^41^,^54^ Although a link between MGMT loss and aberrant expression of p53 is supported by the present findings, it should be noted that only a small number of polyps showed these changes concurrently.

*KRAS* mutation has been linked to the initiation of hyperplastic aberrant crypt foci and small HPs^7^,^38^,^55^ and is therefore closely associated with the development of glandular serration. While the acquisition of *KRAS* mutation is also observed in adenomas, this change is correlated with the development of a villous architecture and in some cases the presence of epithelial serration (see Discussion of Group B serrated polyps above). It may therefore be conceptually correct to view *KRAS* mutation as adding a serrated molecular signature to the traditional adenoma and hence providing an additional ‘fusion’ pathway. However, a mechanistic link between *KRAS* mutation and the morphogenesis of serration and villous change remains to be established. *MGMT* is again implicated in this second type of ‘fusion’ since methylation and inactivation of this DNA repair gene has been linked to G:C to A:T transitions in *KRAS*.^56^–^58^ In this study there was an association between loss of expression of MGMT and *KRAS* mutation among small TAs (*P* = 0.04) but not in the other polyp categories. Methylation of *MGMT* occurs in normal colorectal mucosa,^59^ as well as in polyps, and is therefore unlikely to serve as a key rate-limiting step in the transition to malignancy. A possible third ‘fusion’ pathway implicating *KRAS* and methylation of *APC*^60^ is included in Table 3.

In summary, MPs and SAs account for only about 2% of colorectal polyps. Nevertheless, those serrated polyps with dysplasia show frequent mutation of either *KRAS* or *BRAF* and frequent loss of expression of MGMT (particularly MP). Additionally, four of 25 (16%) showed high-grade dysplasia and in three of these there was concordant aberrant nuclear expression of p53. Along with SSAs, these rare polyps may serve as the precursors of sporadic CRCs with *BRAF* mutation and DNA methylation (with and without DNA MSI) and a subset of CRCs with *KRAS* mutation. Their malignant potential is explained by the accumulation of genetic alterations that may in turn depend upon the inactivation of the DNA repair gene *MGMT*.^28^ The importance of these ‘fusion’ polyps as cancer precursors may be under-appreciated because critical rate-limiting changes governing malignant transition, particularly in association with loss of function of MLH1 of p53, occur rapidly and can rarely be ‘caught in the act’.

## Abbreviations

CRC: colorectal cancer
HP: hyperplastic polyp
MGMT: O-6-methylguanine DNA methyltransferase
MP: mixed polyp
SA: serrated adenoma
SSA: sessile serrated adenoma
TA: tubular adenoma
TVA: tubulo-villous adenoma
VA: villous adenoma
